# Environmental Risk Factors influencing Bicycle Theft: A Spatial Analysis in London, UK

**DOI:** 10.1371/journal.pone.0163354

**Published:** 2016-09-19

**Authors:** Lucy Waruguru Mburu, Marco Helbich

**Affiliations:** 1 Institute of Geography, University of Heidelberg, Heidelberg, Germany; 2 Department of Human Geography and Spatial Planning, Faculty of Geosciences, Utrecht University, Utrecht, The Netherlands; Stony Brook University, Graduate Program in Public Health, UNITED STATES

## Abstract

Urban authorities are continuously drawing up policies to promote cycling among commuters. However, these initiatives are counterproductive for the targeted objectives because they increase opportunities for bicycle theft. This paper explores Inner London as a case study to address place-specific risk factors for bicycle theft at the street-segment level while controlling for seasonal variation. The presence of certain public amenities (e.g., bicycle stands, railway stations, pawnshops) was evaluated against locations of bicycle theft between 2013 and 2016 and risk effects were estimated using negative binomial regression models. Results showed that a greater level of risk stemmed from land-use facilities than from area-based socioeconomic status. The presence of facilities such as train stations, vacant houses, pawnbrokers and payday lenders increased bicycle theft, but no evidence was found that linked police stations with crime levels. The findings have significant implications for urban crime prevention with respect to non-residential land use.

## Introduction

The benefits of cycling to personal health and the environment are well established (see e.g., [1–8]). Subsequently, researchers in the domains of health and transportation have extensively studied the dangers that cyclists face, such as cardiac disease, injury and exposure to carbon monoxide [5,7,9–12]. However, urban authorities grapple with a less explored risk related to cycling, namely theft. A case in point is England and Wales where bicycle theft continues to rise, even while the overall offending rate is declining [13]. The accelerating risk has been linked to the bicycle’s high utility for door-to-door transportation and the relative ease of liquidating it, whole or as parts [14]. Studies show that bicycle owners are three times more likely to lose possession through theft than are owners of automobiles and motorcycles [2,14,15]. Repetitive victimization greatly undermines policies to promote cycling because a large proportion of victims neither replace their stolen bicycles nor resume cycling [2,16,17].

Several measures have been proposed to reduce bicycle theft, such as safer locking mechanisms [2], better parking facilities [18], and interventions that target areas of high risk [15,19]. While these measures might indeed have reduced crime levels, particularly during summer as examined from the data in this study, bicycle theft has not been rigorously analyzed to date [2,14,20]. Research has yet to be undertaken to track where the thefts occurred and investigate the risk factors at these places. Thus, even as the police struggle to control bicycle theft, confidence in their capability to recover the stolen ones continues to decline [21]. For instance, statistics based on the cases that occurred between 2013 and 2016 for Inner London (UK) show that perpetrators of 96 percent of the reported bicycle thefts are as yet unidentified. A major focal concern, therefore, is to anticipate and address the risk factors that increase victimization probabilities in major cities.

This paper’s prime objective is the investigation of potential place-specific factors influencing bicycle theft using police-recorded incidence data. It uses data from London, one of the world’s busiest urban metropolises to address two principal questions:

1. What are the associations between bicycle theft and different public amenities in the urban environment?
2. To what degree are these relationships mediated by the socioeconomic aspects of places?

The paper is structured as follows: First is a review of existing crime literature and a description of the data and methods. Then, results are presented and discussed in terms of policy-related interventions and the paper concludes with a reflection on the possible applications.

## Literature Review

### Theoretical Background

Theories from environmental criminology, such as Routine Activity and Rational Choice [22–24], provide useful clues about why certain places or people might be victimized more than others. Routine Activity Theory is often used to attribute urban crime to the simultaneous coexistence of three elements: 1) motivated offenders, 2) suitable targets, and 3) insufficient guardianship [22,24]. Daily routine activities set the scene for interaction of these elements, and this is why the places that attract multitudes might also present opportunities for crimes such as bicycle theft [23,25]. For instance, field surveys in urban areas have shown that bicycles are commonly stolen from on-street locations [2,16,20] and around facilities such as railway stations and bicycle stands [2,14,19]. In Minneapolis (USA), stores and bars registered the largest number of calls for police service [23], while public transit systems in Los Angeles (USA) suffered significant theft and robbery [26–28]. Places such as these are common points for criminals and their targets to interact. With regard to the extent of such interactions, studies of risk terrain models for crime in New Jersey, USA have uncovered that crimes tend to concentrate over relatively small areas [29,30]. The narrow offender-target activity spaces are also evident in the micro-scale literature in which different types of property crime have been examined over street segments [31,32] and at the house and block levels [33–35]. It might be noted, however, that opportunities that are present in the course of routine activity differ across crime types. For instance, places that record a small number of residents due to extensive outbound commuting tend to experience high burglary rates [36], but thefts are usually more frequent where people abound [34]. Similarly, the large numbers of bicycles near railway stations are bound to attract thieves because railways provide intermediate transportation for many cyclists [17,37].

According to Rational Choice Theory, crime variation is further hinged upon the risks and rewards as well as the offender’s ability to weigh these prior to perpetrating. Sometimes, crimes fail to occur even though offenders interact with targets in the absence of capable guardians because the risk is simply too high. Studies have found out, for example, that police presence can hinder crime considerably [29,38] and that properties of low value are less likely to be stolen than high-priced properties. This is mainly because offenders select alternatives that maximize their expected utility [35]. While Rational Choice Theory has been less successfully used to study patterns of assault and homicide, it is popular for explaining opportunistic crimes such as theft and burglary.

Two ecological theories, namely Social Disorganization and Collective Efficacy, have also been extensively applied in the literature to link crime with the community context [39–41]. Social Disorganization Theory posits that undesirable factors such as residential inequality, economic disadvantage and ethnic stratification disrupt social ties and cause crime levels to increase. Counterpart to Social Disorganization Theory is Collective Efficacy Theory, which links social control and cohesion. This last theory explains why integrated communities are less likely to experience crime. Regression models have been widely used to test these theories against area-based crime concentrations at various spatial scales. Among the epical works is that of Sampson and his colleagues [39], in which fewer crimes were observed in affluent Chicago’s neighborhoods where inhabitants were more united and were more willing to intervene for common safety. Factors such as increased population, residential instability and unemployment were linked with unstable institutions and decreased cohesiveness in the studied areas. A risk analysis for street segments in Philadelphia (USA) could not link ethnic heterogeneity with risk of property crimes, but increased disadvantage was positively related with crime while more affluent streets were found to be less frequently victimized [31]. That being said, both community-specific socioeconomic attributes and place-specific land use features certainly require close scrutiny for security.

### Place-based Risk Assessment

The skew in crime distribution in urban areas can be described using the Pareto principle (“80/20” rule), whereby 80 percent of criminal activity occurs in about 20 percent of public places [30,33,42,43]. An extensive study of robbery in Chicago (USA) revealed that census blocks with public amenities such as bars and clubs, liquor stores, and pawnbrokers suffered more crimes than other blocks [33]. It was further discovered that crime concentration was proportional to distance, whereby robbery counts were often higher near blocks that comprised these amenities while blocks much further away suffered fewer robberies. Findings elsewhere in metropolitan UK show that outdoor theft of properties occurs more frequently in those areas where risk-related facilities are clustered [34]. These observations provide evidence that risk accumulates predominantly because of chronic victimization at places that have particular types of land-use features.

Once the specific risk probabilities for areas are known, strategic interventions could efficiently reduce crime. It is hence crucial to know how different types of urban facilities compare in their levels of dangerousness. However, previous research to that end has generalized the array of non-residential land-use features to categories such as retail and recreation [34]. Although simplification may be inevitable, it can undermine facility-specific risk estimates owing to the complexity of criminal behavior. Additionally, crime investigation needs to account for seasonal variation in offending. This has been partially addressed in the literature on dynamic crime hotspots (see, e.g., [44,45]). For instance, different types of crime have a comparable likelihood of seasonal accumulation in and around transportation hubs [46]. Property vandalism is more prevalent near train stations during winter, while in spring, the areas a little farther away suffer increased theft due to crowding and human interaction [46]. Findings such as these make it important to account for seasonal effects on place-based risk.

The aforementioned studies provide considerable insight, but they are limited in several respects. Firstly, most of these studies have not examined the concentrations of risk for specific types of crime. With a few exceptions (e.g., [34,44,46]), crime outcomes have been aggregated to property crimes and violent crimes prior to risk estimation. It is, nevertheless, insufficient to generalize across types of crime regarding the drivers of urban risk because differences exist in the distribution of activity spaces and opportunities. Thus, the analysis of combined offending behaviors might unveil patterns of crime concentrations, but the place-specific concentrations cannot be linked with individual crimes [47]. For instance, burglary is a crime perpetrated inside buildings and one that tends to draw criminals to particular locales, but crimes such as robbery and bicycle theft usually occur outdoors and are perpetrated at random when opportunities arise [34]. While most forms of theft occur in crowds, some forms, such as purse snatching and pickpocketing, involve direct offender-victim contact while bicycle theft occurs in the victim’s absence. Consequently, even those studies that analyzed theft behaviors (e.g., [34,44]) have left some questions unanswered because they did not examine the distinct behaviors in isolation.

Secondly, the existing literature lacks an accurate description of crime propagation from the physical location of risk-related facilities. A train station located within 100 m of a street segment will most likely pose a different level of bicycle theft risk than another train station located half a kilometer away [31]. Thus, overlooking the proximity of facilities to places as it has been done in the past (e.g., [30,34,48]) can yield unreliable estimates of the risk extent. The interplay of victims, offenders and opportunities varies according to distance and time, and this variation needs to be taken into account in the risk estimation.

The third limitation concerns the generalizability of inferences that are biased towards certain geographic representations of findings. All of the studies cited above, except one (i.e., [34]), were conducted in the USA [30,31,48]. Case studies from other major cities around the globe might broaden the understanding of a notion of risk that is independent of geographic and policy differences.

This paper aims to help fill the research gaps by adopting the case study of metropolitan London to describe the first risk investigation using police-recorded bicycle theft data. The risk models used in this study extend the models that have been successfully applied by Groff and Lockwood [31] with three new features. Firstly, a set of seasonal variables was included to control for offending variation that is due to seasonal changes when examining other structural influences on bicycle theft. Secondly, an offset variable was applied to model the commuter-adjusted population that is exposed to bicycle theft. This took into account the effects of daily movements and interactions of individuals on offending and victimization probabilities. Thirdly, external bicycle theft locations that were within the predefined buffer lengths around Inner London were taken into account in order to avoid border effects.

## Materials and Methods

### Study Area

**Fig 1:**
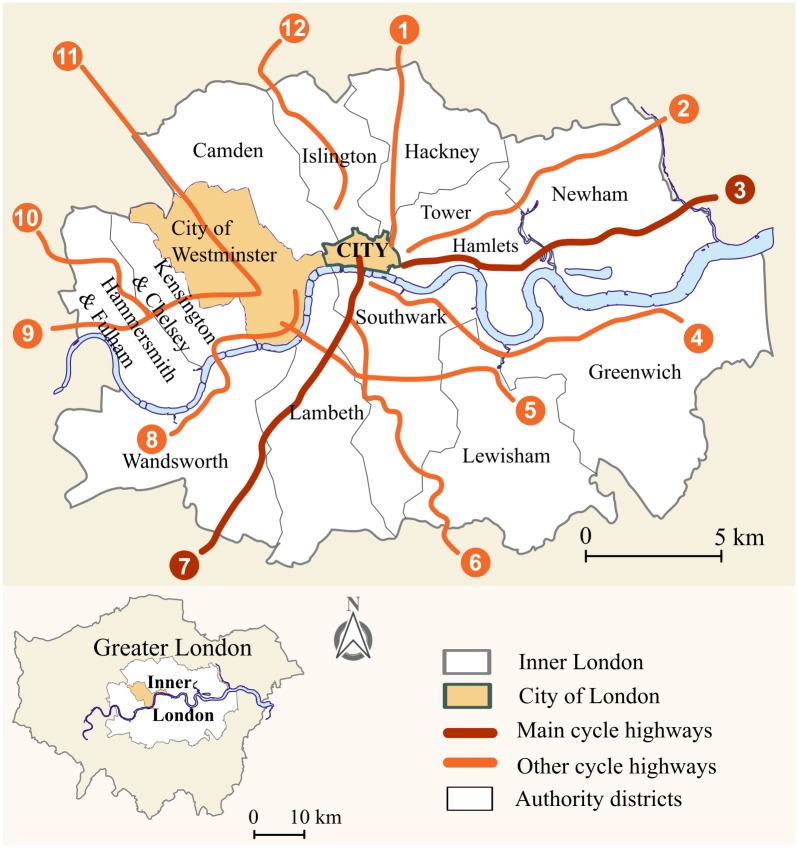
Cycle superhighways in the study area of Inner London.

Bicycle theft is prevalent in London, UK. Although the entire metropolitan region experienced substantial risk, 67 percent of all incidents were reported in Inner London, the study area. It was hypothesized in this study that the interplay of risky places and increased human interactions underlie the crime concentrations. Indeed, Inner London comprises many bars, railway stations and commercial facilities, all of which attract crowds for reasons unrelated to crime but nevertheless provide opportunities for bicycle theft. Inner London is also the location of the City of London, UK’s hub of commercial activity. After the expansion of the infrastructure to include twelve cycle superhighways that interconnect via the City (Fig 1), cycling in London has risen sharply and now registers 177 percent more activity than ten years ago [18]. By extension, the number of possible victims and opportunities for bicycle theft has increased drastically. Another reason to focus on London is the availability of rich sources of administrative data on facility locations, socioeconomic profiles of areas, and crime, a factor that is bound to motivate comparative studies in the future.

### Data

The crime data comprised 36,987 spatially referenced bicycle theft incidents that were recorded over 36 months (between May 2013 and April 2016). Monthly information was deemed sufficient for the risk analysis, particularly because detailed approximation of temporal profiles of property crimes is usually problematic [49]. For example, it may take several days or weeks before a stolen bicycle is reported missing during vacation time.

Locations of public amenities were derived from the Ordnance Survey (UK) and OpenStreetMap databases, depending on data availability. The high volume of contributions from experts and avid users has caused the quality of OpenStreetMap data for London (i.e., accuracy, completeness and consistency) to increase rapidly in the recent years, such that it now compares with that of official administrative data [50,51]. Furthermore, public amenities from OpenStreetMap were matched with Google Maps amenities whenever possible, but no major locational inconsistencies were found.

The network dataset, released by the Ordnance Survey in July 2015, includes 'A' roads that provide large-scale transport links between areas, 'B' roads that feed traffic between ‘A’ roads, other major and minor roads, and the streets that are publicly accessible. Three additional groups of public amenities were obtained from the Ordnance Survey database: 1) universities, 2) train stations, which are potentially criminogenic (see [30,31,52,53]), and 3) police stations, which were hypothesized to mitigate risk [29,38]. Seven types of criminogenic facilities were retrieved from OpenStreetMap database records: 4) bars, 5) pawnbrokers and payday lenders, 6) vacant buildings, 7) cycle renting facilities, 8) cycle stands, 9) cycle repair shops, and 10) street-lining trees. The first four of these seven facility types (i.e., 4–7) have been identified as property-crime generators [30,31,32,48]. Cycle parking and renting locations are hypothesized to generate bicycle theft [2,15,54]. Pawnbrokers and repair shops are crime attractors in the sense that they avail opportunities for disposing of cycle parts [48,55]. The criminogenic influence of street-lining trees is unestablished, but we hypothesized that both the parking convenience and the camouflage that these amenities offer would promote bicycle theft.

Tract-level census data from the 2011 UK census were obtained at the lowest aggregation level. Census data were used to measure social disorganization (i.e., ethnic heterogeneity and deprivation levels) and its mitigating factors (i.e., affluence levels). Additionally, information on the on-street population was obtained by combining data on the residential population with commuting patterns across census areas. The latter corresponds to traffic data that were also obtained from the 2011 UK census on inbound and outbound commuters.

### Determining Risk Exposure

The choice of analysis units is relevant for establishing the structural influences on crime [56]. This study employed 51,216 street segments for the analysis, based on the prior evidence that bicycles are often stolen near streets [15,16]. Buffer zones were calculated for these segments over the road network by measuring the shortest-distance path from the segment’s midpoint outward to each specified buffer distance. Network buffers allow more precise measurements of street-level interactions than geometric buffers [57].

The risk of bicycle theft was operationalized in light of the study region’s spatial configuration and previous studies. While the literature is inconsistent about effective risk-measurement distances, early evidence in Manchester, UK [58] has shown that thieves and shoplifters travel between 640 m and 966 m to offend. More recent studies in Newark and Philadelphia (USA) have identified crime concentrations within 378 m and 120 m of streets respectively [31,48]. We employed suggestions from the literature and identified 40 m (i.e., half the length of a street segment) as the starting point for measuring risk incrementally up to within 1,000 m, the suggested maximum length of offender travel [58]. This resulted in 25 candidate risk models. The underlying rationale was to identify the "true" spatial extent at which risk factors are associated with crime [59]. After evaluating the candidate models for goodness-of-fit using the Akaike Information Criterion (AIC) and examining the statistical significance of predictor variables, four incremental distances measured within 160 m, 320 m, 480 m and 640 m from the street’s midpoint were identified as the optimal distances for risk measurement. The model of the smallest risk measurement distance (i.e., 160 m) was approximately twice the average length of Inner London’s street segments, which is 82.38 m as measured from the study data. In essence, therefore, the selected geographic extents corresponded to two street segments, four segments, eight segments and 16 segments.

### Variables

#### Independent and offset variables

Crimes were linked with street segments using a 160-m-threshold distance along the shortest path of the road network. This linking was performed in a manner that allowed the same crime to be associated with multiple street segments if it occurred within 160 m of each. Restricting the maximum distance for linking crimes allowed to control for the bias of including crimes that have occurred too far away from the streets. While creating risk areas for the numerous segments of Inner London’s streets resulted in overlapped buffers over the street network, each buffer was processed consecutively and independently of any other overlapping buffers. Thus, a single crime in multiple overlapped buffers contributed to the count of each, and crime counts for buffers located beneath other overlapping buffers were unaffected by the overlaps. Additionally, for all the buffers originating from the same street segment’s midpoint, the number of crimes in those buffers measured within 320 m, within 480 m, and within 640 m was the same as the number of crimes in the smallest buffer (i.e., measured within 160 m). Analysis for the larger buffers essentially involved questions such as “how do train stations located until 640 m from here influence the bicycle theft risk for *this* street segment?”

An offset variable, namely Adjusted Population, corresponds to the on-street population that is exposed to risk of bicycle theft. Census-tract-level data on the residential population were adjusted using flow data on everyday commuters in a two-step procedure. First, the Workday Population was obtained using counts of residential population minus outbound commuters plus inbound commuters. Afterwards, weighted counts of Residential Population and Workday Population were combined to estimate the population that would be expected in a certain census block at any particular time. Commuters are expected to be away from their census tracts between 7:00 and 19:00, or between 8:00 and 20:00 on each weekday, an equivalent of 60 hours each week [60,61]. Thus, out of the 168 hours constituting the week, 60 hours were assigned to Workday Population and the remainder (108 hours) to Residential Population. The offset variable, Adjusted Population, was derived as:

$$\text{Adjusted Population }=\text{ ​}\left(\text{Workday Pop}.\times \frac{60}{168}\right)\;+\left(\text{Residential Pop}.\times \frac{108}{168}\right).$$ (1)

Tract-level census data are usually aggregated to areas that can contain multiple street segments. Therefore, values of the offset variable were extracted using area-weighted means for each street segment. These correspond to the proportion of data in a census tract that intersects the road network buffer, assuming a uniform distribution of values within the census tract.

#### Predictor variables

The analysis included 13 risk correlates. The first ten correspond to the presence of the public amenities that have been described above. Given that *d*_*ij*_ is the distance from an amenity to the midpoint of a buffer area and *d*_*max*_ is the maximum distance value, an inverse distance weighting scheme was used to quantify amenity presence within the road network buffers as follows [25]:

$$\text{IDW }=\sum d_{ij}\leq t\left(1-\left[\frac{d_{\text{max}}-d_{ij}}{d_{\text{max}}}\right]\right).$$ (2)

The three other variables correspond to socioeconomic indicators at the census-tract level: 1) Ethnic heterogeneity, 2) Deprivation, and 3) Affluence. As with the offset variable, area-weighted means were employed to configure values of the socioeconomic indicators to street segments. Thus, if a tract has a deprivation level of 0.5 and constitutes half of the road network buffer of immediate focus, it contributes 0.25 to the area-weighted mean deprivation value of the corresponding street segment.

Ethnic heterogeneity employs Blau's index [62] to calculate the proportions of ethnic strata in areas. This is a probability score corresponding to the inverse of summed-up squares for 18 major ethnic subdivisions, i.e.,

$$E=1\;–\sum p_{i}^{2},\;i=\;\left\{1,\;2\ldots ,\;18\right\},$$ (3)

where *p*_*i*_ is the proportion of individuals belonging to an ethnic group and a greater value for *E* corresponds to a more heterogeneous area. Deprivation classifies the proportion of households with at least three of these disadvantages: a) unemployment or chronic illness, b) long-term illness, c) no higher education, and d) living in shared abode and/or with no central heating. Affluence is a composite variable comprising two indicators, namely the proportion of all household reference persons that are middle or top managers and the proportion of outright homeowners. The composite was generated using unit-weighted *z*-scores of the constituent indicators to create a stable measurement (see e.g., [63]).

**Fig 2:**
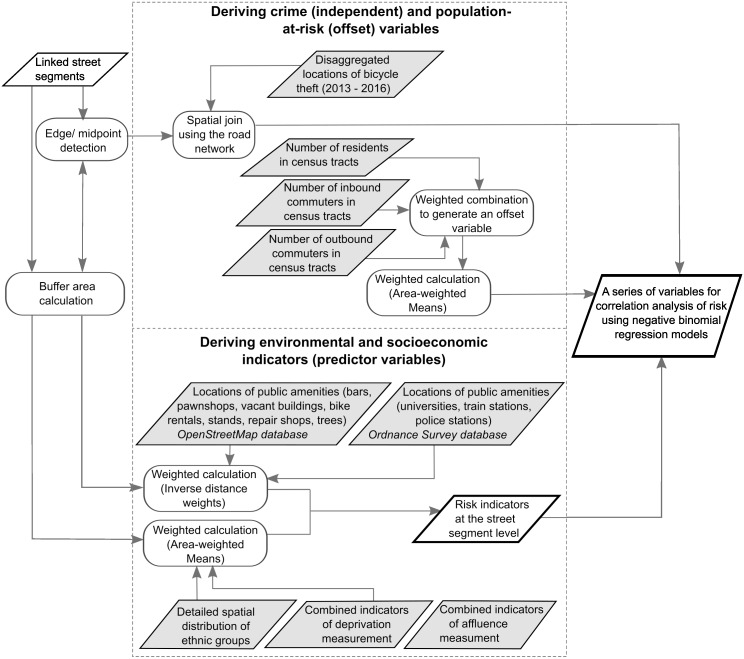
Flowchart of the experimental design to identify risk indicators for regression modeling of bicycle theft.

Seasonal variation in bicycle theft was incorporated into the crime models by including a set of three binary-based (0, 1) dummy variables: Summer, Autumn, and Winter. This controlled for offending variation that is due to seasonal changes and also examined whether the risk in a certain season was any different from that observed in spring. Fig 2 provides a description of how the study variables were derived.

### Method

Owing to over-dispersion in the bicycle theft data (i.e., the mean exceeding the variance [64]), the negative binomial regression was applied as an alternative to the Poisson regression for modeling risk factors [64]. Four models were estimated to take the uncertainty of the spatial context into account. These are based on four incremental threshold distances: 160 m (Model 1); 320 m (Model 2); 480 m (Model 3); and 640 m (Model 4).

Streets are highly interconnected in metropolitan London, and significant spatial autocorrelation in the crime counts makes it necessary to model the spatial structure of offending [53]. Thus, models were adjusted for the theft incidence in the immediate surrounding area. For example, the model of risk within a buffer radius of 320 m incorporates in its spatial lag variable the number of bicycle theft incidents in the surrounding buffer area measured between 321 m and 480 m. All regression models employed standardized predictor variables to increase the comparability of different risk factors.

## Results

### Descriptive Statistics

**Fig 3:**
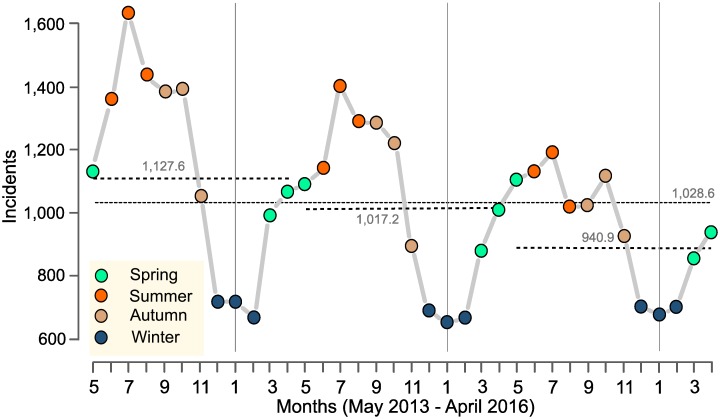
Seasonal bicycle theft statistics for Inner London from May 2013 to April 2016 (*n* = 36,987 events).

About a thousand bicycles per month were stolen from the streets of Inner London each year, with an average monthly incidence of 1,029 crimes being recorded over the three years (Fig 3). Crime peaked in the summer of each year (June–August) and the offending rate was highest in July. However, bicycle thefts decreased from year to year especially during the summer period, a factor that might be due to interventions such as safer locking mechanisms and improved parking facilities [2,18]. The offending rate decreased steadily each year between autumn and winter. The cold winter months (December–February) registered less than half the crime frequency that was experienced in summer. In spring (March–May), crime rates rose steadily and peaked again during summer. This pronounced seasonal pattern made it necessary to adjust for offending seasonality in the regression models.

After bicycle theft events were linked to streets, statistics showed a high offending frequency. The mean count for street segments was 17 crimes and the range was between zero and 330 events as observed from the crime data for the entire observation period. The offending distribution was highly variable. The standard deviation estimate (SD = 21.7) was even higher than the mean and suggestive of over-dispersed observations. Condition indexes [65] indicated multicollinearity of predictors that was solved after removing some variables (e.g., Entertainment facilities and Food places). Table 1 gives the descriptive statistics for predictor variables that were admissible in the negative binomial regression.

**Table 1:** The distribution of risk indicators over Inner London’s street segments (*n* = 51,216 segments).

Variable	Buffer Distance															
	160 meters				320 meters				480 meters				640 meters			
	Min.	Mean	Max.	SD	Min.	Mean	Max.	SD	Min.	Mean	Max.	SD	Min.	Mean	Max.	SD
Bars	0	0.033	4.346	0.176	0	0.107	5.418	0.383	0	0.228	7.865	0.690	0	0.396	10.741	1.109
Bike rentals	0	0.058	2.409	0.195	0	0.188	4.208	0.402	0	0.401	5.866	0.742	0	0.699	8.582	1.231
Cycle stands	0	0.278	12.971	0.715	0	0.852	17.724	1.589	0	1.743	22.287	2.743	0	2.957	32.427	4.197
Cycle repair	0	0.002	1.693	0.037	0	0.007	1.846	0.066	0	0.016	1.966	0.099	0	0.027	2.224	0.134
Pawnbrokers	0	0.036	1.928	0.157	0	0.127	2.295	0.276	0	0.277	2.775	0.381	0	0.491	3.081	0.429
Police stations	0	0.018	2.663	0.115	0	0.061	4.918	0.244	0	0.132	6.894	0.413	0	0.231	8.596	0.625
Trees	0	3.731	547.093	11.853	0	11.808	653.044	32.035	0	24.951	702.235	62.787	0	43.566	960.109	104.870
Universities	0	0.011	5.121	0.109	0	0.032	6.060	0.218	0	0.072	6.679	0.340	0	0.128	7.259	0.469
Train stations	0	0.019	1.193	0.104	0	0.057	1.597	0.175	0	0.199	2.003	0.248	0	0.199	2.502	0.329
Vacant houses	0	0.009	4.844	0.111	0	0.029	5.922	0.203	0	0.060	6.826	0.308	0	0.103	7.913	0.427
Ethnic heterogeneity	0.121	0.717	0.888	0.110	0.196	0.725	0.891	0.104	0.205	0.731	0.893	0.100	0.208	0.736	0.895	0.096
Deprivation	0	0.067	0.248	0.038	0	0.072	0.250	0.037	0	0.075	0.252	0.036	0.001	0.077	0.256	0.035
Affluence	-2.795	0.111	4.051	1.209	-2.599	0.122	4.234	1.284	-2.413	0.131	4.561	1.362	-2.408	0.141	5.074	1.464

As expected, higher values of predictors were registered with each systematic increase in the geographic extent of measurement. Street-lining trees were the most prominent feature among the predictor variables, followed by the ethnic heterogeneity of areas. Bicycle repair shops were the least prominent. However, variation was significant across all variables. All variables had zero as the lowest value for street segments apart from two predictors, namely Ethnic heterogeneity and Affluence.

### Regression

Model 4, which modeled risk exposure within a 640-meter radius of street segment, provided the best fit (see Table 1). It estimated a value of 52,842 on the Akaike Information Criterion (AIC), almost half the score of Model 1, the model measuring risk within 160 m (AIC = 92,349). Additionally, Model 4 had the highest r-square estimate. Predictor variables accounted for 51 percent greater variation in risk with this model (i.e., *R*^2^ = 0.803) than with Model 1 (*R*^2^ = 0.410). Estimates of seasonal dummies and other predictor variables correspond to incidence rate ratios (IRRs), the exponential forms of regression coefficients. The value of the IRR denotes the multiplicative change of influence following a one-unit change in the predictor variable [66]. Accordingly, an IRR value of 1.5 corresponds to 50 percent increase in the likelihood of bicycle theft following a one-unit rise in the standard deviation of exposure values summarized in Table 1. Analogously, IRR = 0.5 indicates that a one-unit increase mitigates risk by half. IRR precision measurements employed 95 percent confidence intervals.

Seasonal effects were generally consistent across the four models. With spring as the reference category, bicycle theft likelihood was lower in winter and higher in summer and autumn. Street segments experienced the highest risk in summer. However, risk likelihood in the autumn season was positive when measuring out within the first three distances of street segments, but it was negative within 640 m. All seasonal estimates were statistically significant at the *p* < 0.001 level, and their confidence intervals indicated high precision.

**Fig 4:**
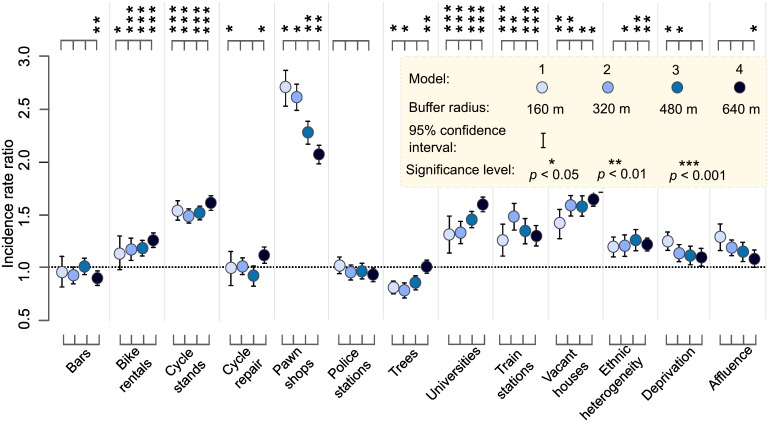
Incidence rate ratios (IRRs) and 95% confidence intervals of negative binomial models. Estimates correspond to effects of risky and risk-mitigating amenities and socioeconomic factors on bicycle theft. Effects are measured using bicycle theft counts (May 2013 to April 2016) for 51,216 street segments. Models account for the seasonal effects shown in Table 2 and assess risk exposure over four threshold distances: (a) 160 m—Model 1; (b) 320m -Model 2; (c) 480m—Model 3; and (d) 640m—Model 4. The commuter-adjusted population is modeled as an offset variable.

Fig 4 presents risk estimates for 13 variables across all four cumulative distance-based models. All the risk factors were estimated with high precision, but about half of the public amenities had broader confidence intervals (i.e., lower precision) when measuring out within 160 m of street segments owing to the smaller number of these amenities at this distance. Six out of ten categories of amenities were significantly correlated with bicycle theft at all distances, even after adjusting the models for seasonal variation and socioeconomic status. With respect to effect sizes, amenities such as bicycle stands, pawnshops, universities, train stations and vacant houses had greater IRRs than any of the socioeconomic variables. Pawnbrokers and payday lenders posed by far the highest risk of bicycle theft. Their representative variable, Pawnshops, had a risk estimate of 2.71 within 160 m, which was more than three times the estimate for most other variables. Even though the effect size decreased with the measurement distance, the risk influence of pawnshops within 640 m was still greater than that of other variables (IRR = 2.12, *p* < 0.01).

The risk-aggravating influence of bicycle stands and universities was highly significant at all distances (at *p* < 0.001), and their IRR values accrued generally with increasing distance. This trend whereby the magnitude of influence increases with distance was common for many variables. Among the public amenities, bars, cycle repair shops and police stations had the least amount of influence both in magnitude and significance. Bars influenced a 12 percent risk reduction within 160 m (IRR = 0.88, *p* < 0.01). Similarly, trees had a risk-mitigating influence when measuring outwards within 160 m and within 320 m from the street segment’s midpoint. The influence of trees within 640 m was significant (*p* < 0.01), albeit at zero (i.e., IRR = 1). Bicycle repair shops also exerted no influence within 160 m, but were moderately criminogenic within 640 m (IRR = 1.13, *p* < 0.05). Police stations did not affect risk variation at any measurement distance.

Socioeconomic variables were generally less significant to bicycle theft than public amenities. Only the Deprivation variable influenced risk within 160 m of street segments (IRR = 1.23, *p* < 0.05). Deprivation and Ethnic heterogeneity were criminogenic within 320 m, while only the latter variable posed risk within 480 m. The Affluence variable had no significant influence, except when measuring out within 640 m. Even at this distance, the effect was negligible at 11 percent (IRR = 1.109, *p* < 0.05).

Including the spatial lag variables appears to have effectively modeled residual spatial autocorrelation. Moran’s *I* statistics of the residuals across models 1 to 4 were not significant on the *p* = 0.05 level.

## Discussion

This study has investigated the risk that emanates from urban land-use features to provide grounds for security-directed intervention on bicycle theft. Risk analysis was performed over road network buffers instead of the conventional census areas, a choice consistent with the knowledge that most cycle thefts are on-street incidents [15,16]. A novel exposure variable was included to model the on-street target population, which took into account patterns of everyday mobility.

### Key Findings

Risk was deviant across variables and the way exposure was being measured over street segments. However, there was a trend of risk accruing with each increment in exposure distance, particularly for public amenities. All the estimates but two (i.e., for police stations and deprivation) were statistically significant within 640 m of street segments, confirming the high goodness-of-fit statistics for this model (Table 2).

**Table 2:** Effect estimates of negative binomial bicycle theft models for the seasonal variables ^a^ at four threshold distances of risk exposure measurement (models are adjusted for risk factors).

	Model 1 (160 m)			Model 2 (320 m)			Model 3 (480 m)			Model 4 (640 m)		
Variable ^b^	IRR	95% C.I. lower	95% C.I. upper	IRR	95% C.I. lower	95% C.I. upper	IRR	95% C.I. lower	95% C.I. upper	IRR	95% C.I. lower	95% C.I. upper
Intercept	0.013***	0.012	0.014	0.016***	0.014	0.018	0.016***	0.015	0.017	0.010***	0.009	0.011
Winter	0.802***	0.791	0.812	0.779***	0.767	0.790	0.736***	0.725	0.746	0.668***	0.658	0.678
Summer	1.804***	1.791	1.818	1.856***	1.841	1.872	1.929***	1.912	1.947	1.744***	1.729	1.759
Autumn	1.063***	1.050	1.077	1.376***	1.335	1.418	1.136***	1.120	1.152	0.975***	0.962	0.989
Dispersion *theta* ^c^	14.560***			15.57***			19.786***			16.412***		
Nagelkerke *R*^2^	0.410			0.653			0.691			0.803		
AIC	92,349			79,582			71,240			52,842		

Notes:

*** *p* < 0.001.

^a^ Models incorporate seasonality using three dummy variables.

^b^ Regression models control for predictor variables listed in Table 1 and employ the commuter-adjusted population as offset.

^c^ The parameter, *theta* indicates the amount of adjustment for over-dispersion in the Poisson model.

Three observations contribute new evidence to the existing criminological literature. Firstly, studies have traditionally relied on census-based socioeconomic indicators to quantify crime risk in given areas [36,41,52,66–68]. The findings here, however, show that public amenities are far more profound determinants of risk. After adjusting for the effects of seasonal variation and examining all the variables comparatively, more than half of the public amenities had higher risk estimates than socioeconomic indicators. With respect to outcomes of socioeconomic variables, affluence in areas did not impact crime levels except when measured within 640 m. Moreover, the positive influence at this distance contradicts the literature that has always applied Collective Efficacy Theory to classify affluent areas as being safe (see e.g., [32,36,39,40,61]). The difference in observations here possibly owes to the presence of high-priced bicycles in such areas which attract criminals, as consistent with Rational Choice Theory [22,35].

Secondly, we found no influence of police stations on bicycle theft at any distance. This finding contradicted our hypothesis, given that police stations have been linked with crime reduction in the past [29,38]. It seems that a number of crime-specific factors could be overriding this safety effect, such as the lack of direct offender-victim contact and the delay in discovering bicycles as missing.

The effect of bars on bicycle theft was generally not significant, but these amenities influenced risk reduction within 640 m (at *p* > 0.01). This contradicts the criminological literature that has extensively linked bars with increased risks of property crimes and violence [46,31]. Routine Activity Theory provides two possible explanations for this difference. First, the decrease in cycling activity by alcohol consumers may reduce the opportunities for bicycle theft. Second, clients who were frequently exiting the bars to smoke may unwittingly provide guardianship. Further investigation of this outcome is, nevertheless, necessary to shed more light. Overall, our observations suggest that results of risk measurements and predictions using other types of crimes might be ineffective for bicycle theft intervention.

The results of this study also supported the theoretical knowledge that risk stems from certain elements of the physical environment [22,24]. For example, train stations, pawnshops, universities and vacant houses influenced risk at all distances, in line with the literature linking these types of amenities with increased crime levels [31,33,46]. Pawnbrokers are hypothesized to provide opportunities for liquidating stolen property [48,55,67]. In this regard, pawnshops represent places that generate risk because of illegitimate activities that attract both offenders and citizens. Vacant houses have also been linked with property crimes in the past [38,66,67]. It is possible in this case that vacant buildings are provisional stores for stolen bicycles. Nevertheless, the literature linking universities (or learning institutions) with property crimes has generally reported variable outcomes (e.g., [31,52,68]), and further investigation is needed.

Among the risk factors that have not been elucidated in the criminological literature, bicycle renting places, parking stands and repair shops contributed significant risk for bicycle theft. The criminogenic influence was particularly obvious within the largest distance of risk measurement. Evidence of insecure locking practices has tied bicycle theft to parking stands [2,16], and the high precision of the estimation confirmed this criminogenic influence. Similarly, renting shops warrant security prioritization. Risk influence was less noticeable for repair shops, particularly within close proximity to the street segments. Nevertheless, the positive risk influence within 640 m suggests that repair shops are potential venues for selling stolen cycle parts.

The effect of trees (or the natural vegetation) on crime levels has also not been examined in the past, but we expected that trees avail inadequate mechanisms for securing bicycles in addition to concealing a stealing act from the guardians. Previous findings have indeed linked bicycle theft with poor locking practices [2,16]. Instead, this study identified a negative effect of trees that was robust to control for both the socioeconomic status of areas and other amenity-based influences. In the same vein, it can be noted that after disaggregating the event data according to seasons, the lowest risk was estimated during winter and highest during summer as consistent with findings from previous analyses of seasonal crime [44,45]. We thus suppose that the attraction of street-lining trees to walkers and idlers during summer might have discouraged bicycle thieves in certain areas because of the increased risk of apprehension.

### Strengths and Limitations

This study is the first investigation of risk exposure for cyclists that assesses the actual locations of crimes. The past research has contributed to this topic by analyzing survey data [2,15]. By modeling risk factors for bicycle theft over road network buffers, this study offers a more realistic approach to crime investigation than studies utilizing census areas. The second key strength of this risk analysis is that it explores a rich set of area-based urban characteristics, including both the community context and the distinct features of land use in targeted areas. The community context has been highlighted in many studies (e.g., [25,61,68]). However, urban land use turned out to have more influence on the risk of bicycle theft, in line with similar observations that propose criminogenic places to be more finely-distributed than the broader area-based socioeconomic risk factors [30,48]. The third strength of this study is that by considering risk factors over different levels of geographic extent, it could identify objectively measured exposure to bicycle theft, while also accounting for the influence of seasonal variability in risk.

Among the limitations, this study was hampered by the large proportion of bicycle thefts that often go unreported [23,43,49]. Nonetheless, administrative police records correspond to a significant sample of the crime statistics in London, and the results reported here certainly break new ground. Secondly, even though this analysis examined seasonal crime influences, it did not consider the finer temporal rhythms such as daily, weekly, and hourly crime distributions, which can provide relevant clues to assess bicycle theft risk. Thirdly, the data referred specifically to central London. Neither the far-reaching spatial influences of bicycle theft risk across Greater London nor the potential interaction effects across multiple urban regions were examined. Because of the high accessibility between regions in established urban areas such as London, the spatial extent of risk across regions should certainly be investigated. For instance, a study might chart the inter-regional distribution of bicycle thefts over cities that are linked by railway lines.

Given these limitations, further research is necessary. First, risk investigators could tap into reports about bicycle theft from the social media and “lost-and-found” websites, authoritative or otherwise, thus increasing representation of the crime statistics. Effects of land use can also be examined at a finer temporal granularity to understand the differences in the distribution of risk effects by day versus night, or to compare weekends with weekdays. Furthermore, the scarcity of information about how risk factors are distributed with respect to bicycle theft calls for subsequent investigation in other established cities, but also in newly industrialized and developing cities and countries.

### Implications

The major policy implication of this research is that law enforcers and policymakers can focus on certain aspects of the physical environment to strategically prevent bicycle theft, given the well-known difficulty of solving this type of crime after it has occurred. With respect to the facilities directly associated with mobility, or to the linkage of different mobility systems, both train stations and bicycle parking stands have been shown to pose high risk for bicycle theft. Such places need to be evaluated, keeping the seasonal variation and the crime problem in surrounding areas in mind. Other amenities unrelated to mobility but nevertheless availing interaction and opportunities for bicycle theft also require security prioritization, such as universities, vacant houses and street-lining trees.

When considering where and how to prevent bicycle theft, the exposure to risk factors within the physical environment might have profound implications for developing strategic prevention mechanisms within small areas. These environmental factors might be more pertinent than the sociodemographic composition, which is usually quantified over larger and arbitrarily defined areas.

Correspondingly, ethnographic evidence has revealed that even in the census blocks with crime-aggravating socioeconomic influences, crime is not ubiquitous [48]. Such an observation challenges us to look beyond areas that are contextually meaningful to identify factors that could influence crime, factors other than those conventionally measured along with area-based socioeconomic status.

## Conclusion

This paper has examined in detail the influence of different urban facilities on place-specific risk of bicycle theft. It constitutes the first incidence-based bicycle theft risk analysis, which also concurrently assesses amenity-related and socioeconomic influences while adjusting for seasonal offending variation. In tandem with the differences in offending behaviors and opportunities that set bicycle theft apart from other crimes, the unique outcomes that were observed in this study contribute significant evidence to the literature on victimization.

The findings also highlight the important contribution of urban facilities to crime statistics. Even after controlling for seasonal variation and the principal socioeconomic area-based components, effects of urban land use are important predictors of the risk of bicycle theft. Specifically, since the findings indicate that not all facilities pose the same level of risk, risk-estimation models should depict the cumulative weighted influence of nearby facilities. The results could also be used to support the promotion of attributes that are associated with a low risk of bicycle theft.
